# The Impact of Artificial Intelligence on Microbial Diagnosis

**DOI:** 10.3390/microorganisms12061051

**Published:** 2024-05-23

**Authors:** Ahmad Alsulimani, Naseem Akhter, Fatima Jameela, Rnda I. Ashgar, Arshad Jawed, Mohammed Ahmed Hassani, Sajad Ahmad Dar

**Affiliations:** 1 Medical Laboratory Technology Department, College of Applied Medical Sciences, Jazan University, Jazan 45142, Saudi Arabia; aalsulimani@jazanu.edu.sa (A.A.); mhassani@jazanu.edu.sa (M.A.H.); 2 Department of Biology, Arizona State University, Lake Havasu City, AZ 86403, USA; naseem.akhter@asu.edu; 3 Modern American Dental Clinic, West Warren Avenue, Dearborn, MI 48126, USA; fatimajameela9@gmail.com; 4 College of Nursing, Jazan University, Jazan 45142, Saudi Arabia; rnashgar@jazanu.edu.sa (R.I.A.); arshadjawed29@gmail.com (A.J.)

**Keywords:** antimicrobial, diagnostic, monitoring, outbreak, surveillance

## Abstract

Traditional microbial diagnostic methods face many obstacles such as sample handling, culture difficulties, misidentification, and delays in determining susceptibility. The advent of artificial intelligence (AI) has markedly transformed microbial diagnostics with rapid and precise analyses. Nonetheless, ethical considerations accompany AI adoption, necessitating measures to uphold patient privacy, mitigate biases, and ensure data integrity. This review examines conventional diagnostic hurdles, stressing the significance of standardized procedures in sample processing. It underscores AI’s significant impact, particularly through machine learning (ML), in microbial diagnostics. Recent progressions in AI, particularly ML methodologies, are explored, showcasing their influence on microbial categorization, comprehension of microorganism interactions, and augmentation of microscopy capabilities. This review furnishes a comprehensive evaluation of AI’s utility in microbial diagnostics, addressing both advantages and challenges. A few case studies including SARS-CoV-2, malaria, and mycobacteria serve to illustrate AI’s potential for swift and precise diagnosis. Utilization of convolutional neural networks (CNNs) in digital pathology, automated bacterial classification, and colony counting further underscores AI’s versatility. Additionally, AI improves antimicrobial susceptibility assessment and contributes to disease surveillance, outbreak forecasting, and real-time monitoring. Despite a few limitations, integration of AI in diagnostic microbiology presents robust solutions, user-friendly algorithms, and comprehensive training, promising paradigm-shifting advancements in healthcare.

## 1. Introduction

Microbial diagnosis, a crucial component of healthcare, involves characterizing microorganisms through methods like molecular analysis, culture, and imaging. However, traditional procedures encounter difficulties in sample handling, culture issues, misidentification, and delays in antimicrobial susceptibility testing [1]. The utilization of artificial intelligence (AI) in healthcare is swiftly revolutionizing conventional techniques in many fields, such as microbiological diagnostics. Microbiology faces the task of finding and fighting infectious diseases. AI provides a promising solution by providing sophisticated computational tools that can analyze intricate microbiological data quickly and accurately [2].

The progress made in AI, namely in the fields of machine learning (ML) and deep learning (DL), has greatly contributed to the creation of new and inventive methods for diagnosing microbial infections [3,4]. These technologies allow for the quick analysis of various datasets that include genetic sequences, microbiological phenotypes, and clinical metadata. This makes it easier to identify subtle patterns and connections that may go unnoticed by humans. Through the utilization of AI, healthcare practitioners can accelerate the process of diagnosing medical conditions, resulting in prompt interventions and enhanced patient outcomes. AI-driven algorithms swiftly analyze extensive datasets, facilitating the rapid detection of infections and improved treatment strategies [5].

Furthermore, the use of AI in microbiological diagnosis has significant potential for personalized medicine, enabling customized treatment approaches based on unique patient profiles. AI utilizes data analysis, pattern recognition, and diagnostic processes to enable early disease identification, advancements in treatment, individualized treatment plans, and epidemic surveillance [5]. This shift in paradigm not only improves the effectiveness of antimicrobial treatments but also tackles the growing problem of antimicrobial resistance (AMR), which is a crucial concern in modern healthcare [6].

This review examines the growing influence of AI on the diagnosis of microbial infections. It explores the various ways AI is being used, the advantages it offers, and the difficulties it presents, all within the framework of the most recent research and technological progress. The latest research and advancements available in AI-driven diagnostics are used to provide a detailed understanding of how AI is changing the field of microbial diagnosis and creating new methods for fighting infectious diseases.

### 1.1. AI and Machine Learning in Human Health

AI holds the capacity to provide accurate, personalized diagnoses while ensuring the protection of patient rights and enhancing accessibility to medical devices [7,8]. AI, exemplified by ChatGPT, is becoming increasingly pervasive in everyday life, with over 100 million active users [9]. Laymen can now interact with powerful AI tools without prior technical knowledge, and 80% of Fortune 500 companies have integrated ChatGPT into their operations [10,11]. Next-generation AI and ML models allow for the exploration of disease-related datasets, uncovering hidden patterns, including critical regions within multi-omics sequencing data. ML involves fitting mathematical models to data to reveal relational patterns, providing insights into complex biological systems underlying various diseases [12]. It encompasses supervised learning, using labeled data for training, and unsupervised learning, uncovering intrinsic patterns in unlabeled data through clustering.

Deep learning, a subset of ML, relies on artificial neural networks (ANNs), similar to biological neural networks. ANNs consist of interconnected nodes arranged in layers, processing input data to yield predictions. DL holds promise for advancing understanding and predictions in various domains, contingent upon the availability of high-quality data. A powerful synergy between computational prowess and human-like decision-making is emerging in AI and ML, promising tangible impacts on human health. ML has the potential to influence microbial diagnostics, particularly through its integration with biomedical data. Predictive algorithms leveraging ML are revolutionizing disease detection, personalized medicine, and cancer diagnosis and prognosis [13]. By incorporating DL into digital image analysis, significant advancements in computational pathology have been achieved, enhancing the detection of cancer metastases in lymph nodes [14], scoring Ki67 in breast cancer [15], grading Gleason in prostate cancer [16], and predicting molecular marker status [17]. These applications have significantly boosted the accuracy of predicting cancer susceptibility, recurrence, and mortality by 15–25% [18]. The fusion of AI and the Internet of Medical Things (IoMT) is yielding next-generation intelligent healthcare solutions, exemplified by efficient point-of-care biomedical systems. AI’s support for advanced robotic surgeries and the enhancement of IoMT device functionality underscore its transformative potential in healthcare [19]. Additionally, AI-driven techniques are revolutionizing colorectal cancer diagnosis and treatment [20], contributing to individualized care like computer-assisted drug delivery techniques [21].

The human body produces a range of volatile organic compounds (VOCs) as part of its metabolic processes, which can be detected in bodily fluids such as blood, breath, or faeces [22,23]. Changes in VOC profiles have been linked to various diseases, including cancer [24], pneumonia [25], tuberculosis [26], and coeliac disease [27]. Pathogenic microorganisms, including bacteria and fungi, also emit VOCs, which play roles in communication, growth regulation, and pathogenicity [28,29]. Despite the potential for VOCs to serve as biomarkers for infectious diseases, identifying specific VOC signatures associated with microbial pathogens remains a challenge. Early studies have utilized sensitive analytical techniques like gas chromatography coupled with mass spectrometry (GC-MS) to analyze VOCs emitted by microorganisms [30,31,32]. However, the vast amount of data generated from different studies makes interpretation challenging, hindering the establishment of consistent VOC–microorganism associations. ML approaches, including unsupervised methods, have been used to analyze VOC datasets, revealing similarities in VOC patterns between pathogenic and non-pathogenic microorganisms [33,34,35,36]. Recently, a comprehensive dataset of VOCs released by microbial agents from reports published between 1977 and 2016 was compiled, and supervised ML methods were applied to discriminate human pathogen species based on VOC patterns [37,38]. The findings indicate that ML techniques, particularly support vector machines (SVM) and feature selection, may accurately identify subsets of microbial VOCs and discriminate between relevant microbial pathogens [39]. The results provide valuable insights for the development of gas-sensing diagnostics capable of detecting infectious agents rapidly and non-invasively, potentially revolutionizing clinical diagnostics.

Predicting AMR through ML also employs a supervised learning method. This involves training the model on a dataset containing labeled information indicating whether bacterial pathogens are susceptible or resistant to antibiotics. During training, the model learns to recognize patterns within features such as gene sequences or minimum inhibitory concentration (MIC) levels, enabling it to accurately predict AMR phenotypes [40]. A pan-genome approach used ML techniques to characterize antibiotic-resistant strains of *Escherichia coli* [41]. This involved identifying core gene clusters and antibiotic resistance genes (ARGs), leading to improved prediction accuracy for AMR genes within the accessory pan-genome. By implementing a genetic algorithm (GA) within their model, the report discerned the presence/absence patterns of gene clusters, discriminating between them through binary representations. The fitness function, based on the area under the curve (AUC), facilitated the GA process in identifying subsets of gene clusters associated with AMR phenotypes, thus enabling better prediction of resistance profiles.

Another approach involved developing an SVM ensemble trained using the pan-genomes of *Staphylococcus aureus*, *Pseudomonas aeruginosa*, and *Escherichia coli* [42]. Similar to the previous model, a binary labeling strategy was employed, resulting in a sparse binary matrix representing the presence or absence of each gene and allele. By encoding these features into a binary matrix, the SVM ensemble could predict AMR phenotypes for a given genotype. The study highlighted the effectiveness of this approach in identifying resistance determinants at the gene level, with a detailed analysis revealing perfect segregation of fluoroquinolone’s resistance profile based on the presence or absence of known AMR-conferring mutations. Through this method, 25 candidate AMR-conferring genetic features were identified, showcasing the potential of ML in elucidating the genetic basis of AMR.

### 1.2. AI Integration in Microbial Diagnosis

Traditional microbial diagnosis faces challenges throughout the diagnostic process, from sample collection to identification and susceptibility testing. Issues arise from improper sample handling, potentially leading to inaccurate outcomes [43]. Culturing and isolating microorganisms can be time-consuming and prone to false negatives, complicated further by antibiotic presence in clinics [1]. The delay inherent in traditional microbial diagnosis can allow diseases to progress, contributing to antibiotic resistance through empirical therapy [44]. Challenges include cross-contamination, false positives, and the lack of real-time data for effective surveillance. Furthermore, the absence of standardized procedures in diagnostic labs adds to the complexity and inconsistency of results [45].

**Figure 1:**
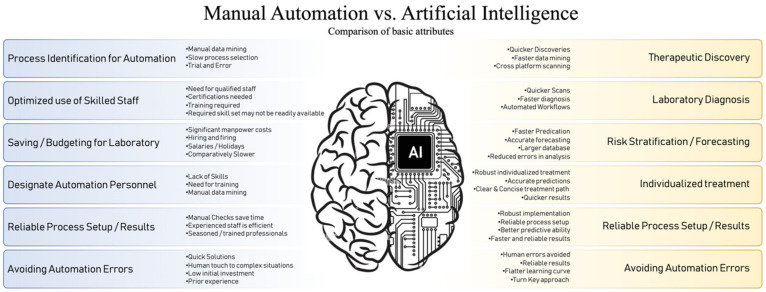
Manual Automation vs. Artificial Intelligence (AI): Automation improves efficiency but requires changes to the laboratory infrastructure and a shift in worker training needs. Utilizing AI on large clinical datasets generated through improved automation will lead to the development of innovative diagnostic and prognostic models. Automation and AI will enable the shift towards personalized medicine.

AI integration in microbial diagnosis has significantly transformed the area by providing quicker and more dependable outcomes in comparison to conventional techniques [46]. AI is highly proficient in the analysis of genetic data, which includes tasks such as identifying pathogens, predicting drug resistance, and uncovering new microbial species [47,48]. ML accelerates the process of analyzing data, resulting in enhanced speed and precision [49]. Conventional microbiological diagnosis encounters difficulties throughout the process, from collecting samples to conducting susceptibility testing. These problems can lead to erroneous results, primarily caused by inappropriate treatment of the samples [50,51,52,53]. Furthermore, the occurrence of delays in diagnosis is a contributing factor to the course of diseases and the development of antibiotic resistance, as indicated by studies [54,55,56,57]. AI integration offers expedited and dependable outcomes via genetic analysis and ML [8,46,58,59]. AI algorithms rapidly and accurately detect patterns and anomalies in large microbiological datasets, which is essential for the early identification of infectious diseases and the development of efficient containment measures [46,60]. Predictive modeling in AI involves utilizing past data to predict microbial activity, which helps in anticipating disease outbreaks, comprehending patterns in antibiotic resistance, and optimizing treatment methods [61]. AI can assist in customizing treatment regimens by considering unique patient features and microbiological profiles, resulting in treatment tactics that are more tailored and successful. ML models, such as DNA sequencers, provide predictions about the genomic sequences of bacteria and viruses, helping clinicians to choose appropriate treatment options [62]. AI facilitates automation, leading to decreased turnaround times and enhanced patient care [59,63]. In general, AI has the capacity to completely transform microbiological diagnostics by offering quicker, more precise, and effective methods that can enhance patient results and alleviate the difficulties linked to conventional diagnostic methods (Figure 1).

This review details the wide-ranging uses of AI in microbial diagnosis within healthcare. Through analysis of recent progress, the review underscores AI’s pivotal role in driving significant improvements in diagnostic precision and expediting progress in microbiology.

## 2. The Impact of AI and Convolutional Neural Networks on the Diagnosis of Various Infectious Diseases

### 2.1. SARS-CoV-2

**Figure 2:**
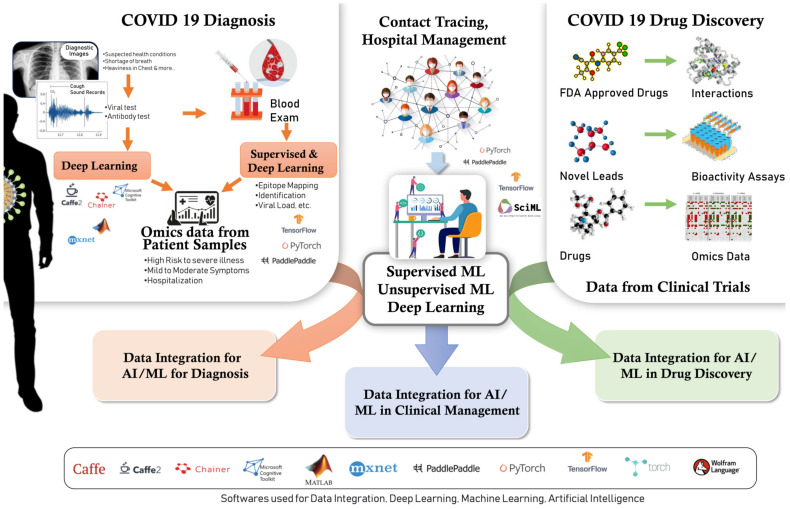
Artificial Intelligence (AI) for the COVID−19 pandemic: Prediction and monitoring involves anticipating the patterns of disease distribution and pinpointing specific areas where outbreaks are likely to occur. Diagnosis and detection will aid in the identification of the virus via image recognition and chatbot-based tests. Drug and vaccine development enhances the speed of identifying new drugs, designing vaccines, and analyzing clinical trials. Resource allocation maximizes the efficient distribution of healthcare resources, such as hospital beds and personal protective equipment (PPE). Contact tracing involves the use of AI-powered systems to track and inform individuals about their exposure risks. Public health surveillance involves the systematic monitoring of public mood, the identification of misinformation, and the tracking of compliance with guidelines. Remote monitoring and telemedicine facilitates the provision of remote patient monitoring and telemedicine services for individuals diagnosed with COVID-19.

DL algorithms, with a special emphasis on convolutional neural networks (CNNs), mark a significant advancement in the field of AI [64]. These sophisticated algorithms exhibit intricate multilayered networks that draw inspiration from the interconnected nature of the human optical cortex. This unique characteristic positions them as a revolutionary tool, particularly excelling in tasks related to imaging and data acquisition along with analysis [64]. In the context of the SARS-CoV-2 pandemic, the pivotal role played by AI and CNNs has been increasingly evident across a spectrum of applications. These applications extend to crucial areas such as virus genome sequencing [65], the formulation of decision-making algorithms [66], drug discovery [67], and vaccine development [68,69]. Noteworthy is the integration of AI and CNNs into pathology laboratories, where digital pathology systems equipped with AI software have become instrumental in elevating the precision of identifying microorganisms present on cytological and histological slides [69]. The benchmark for diagnosing COVID-19 is the detection of the causative agent, severe acute respiratory syndrome coronavirus 2 (SARS-CoV-2), using a virus-specific reverse transcriptase polymerase chain reaction (RT–PCR) test [70]. Along with the rapid spread of COVID-19 through person-to-person transmission during the pandemic came a huge demand for PCR testing in diagnostic laboratories worldwide. To address this, several AI-aided detection models have been developed to enhance the speed and reliability of SARS-CoV-2 diagnosis via RT-PCR. An early study used qPCRdeepNet, a DL model employing a deep CNN to analyze fluorescent readings acquired during COVID-19 RT-PCR, enhancing specificity by identifying false positive results [71]. Another DL model based on the long short-term memory (LSTM) method uses raw fluorescence data from each cycle of RT-PCR testing to shorten diagnosis time, especially when considering patient clinical characteristics and imaging data [72]. Similarly, an AI-based detection and classification system for COVID-19 RT-PCR diagnosis using fluorescent data and amplification curves demonstrated the ability to automatically categorize RT-PCR data [73]. Furthermore, ML models have been used to analyze RT-PCR curves, identifying atypical profiles indicative of contamination or artifacts, thus facilitating rapid and accurate diagnosis while minimizing false positives [74]. ML algorithms have also been used to detect SARS-CoV-2 variants based on cycle threshold (Ct) values in RT-PCR data [75], while the dense neural network (DNN) algorithm can be used for the same using biosensing platforms [76]. Furthermore, many studies have used ML for predicting SARS-CoV-2 positivity based on patients’ blood test and serum profiling results. A random forest algorithm trained with blood test results achieved a diagnostic accuracy of 81% [77], while other ML models using hematochemical values report accuracies ranging from 82% to 86% [78,79]. Moreover, AI has been employed for analyzing protein profiles [80], metabolomics [81], and serological signatures [82] related to SARS-CoV-2 infection using various techniques such as matrix-assisted laser desorption ionization–time of flight mass spectrometry (MALDI-TOF-MS) and liquid chromatography–mass spectrometry (LC-MS-MS). In symptomatic patients suspected of SARS-CoV-2 infection, chest-computed tomography (CT) plays a vital role in diagnosis and isolation. AI has been extensively used for the automated analysis of chest X-ray (CX-R), CT, and ultrasound images to aid radiologists and specialists in decision making [83]. Transfer learning and CNNs applied to CX-R images [84] and chest CT, respectively, have shown high accuracy, sensitivity, and specificity of COVID-19 diagnosis [85,86]. Few systematic reviews on AI-assisted chest imaging for COVID-19 and pneumonia diagnosis with DL models show high diagnostic performance [87,88,89,90]. A recent meta-analysis analyzed a large dataset comprising 51,392 confirmed COVID-19 patients found Resnet to have the best performance, with a sensitivity of 0.91, specificity of 0.90, and AUROC (area under the receiver operating characteristic) of 0.96 [91]. High sensitivity, specificity, precision, accuracy, and F1 score for AI techniques in COVID-19 diagnosis have been reported [92]. These AI tools proved crucial for the early identification and isolation of infected individuals, aiding in pandemic control efforts [91] (Figure 2 and Table 1).

### 2.2. Malaria

**Figure 3:**
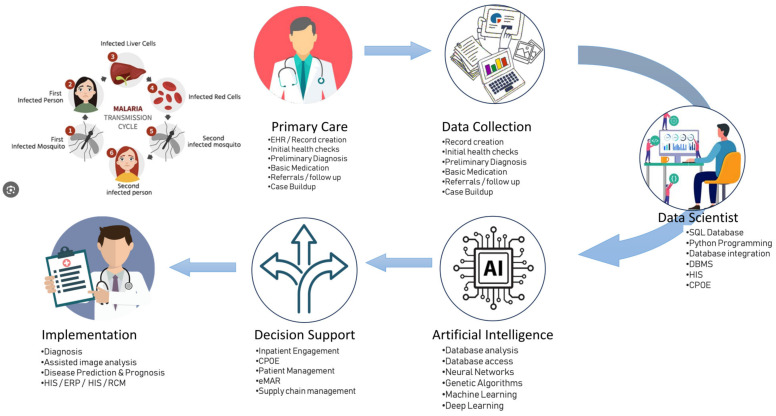
Artificial intelligence (AI) contributes to the fight against malaria by forecasting outbreaks, identifying infections through the analysis of images, tracking treatment resistance, improving methods to manage disease-carrying vectors, expediting the discovery of new drugs, and assisting in the planning of public health initiatives. The system examines many data sources to forecast outbreaks, facilitates precise diagnosis from blood samples, monitors drug-resistant strains, enhances vector control techniques, expedites medication research, and aids in allocating resources for successful treatments. EHR: electronic health record, SQL: structured query language, DBMS: database management system, HIS: health information system, CPOE: computerized physician order entry, eMAR: electronic medication administration record, ERP: enterprise resource planning, RCM: revenue cycle management.

Malaria, among the myriad infectious diseases, stands as a global health challenge with unprecedented impact. The year 2020 alone witnessed a staggering statistic, with over 240 million individuals diagnosed with malaria, leading to a grim toll of more than 600,000 lives lost [98,99]. Swift and accurate diagnosis is paramount for effective therapy in the case of malaria. Traditional methods involve peripheral blood smears (PBS) and manual identification of parasite-infected red blood cells through light microscopy (LM), considered the gold standard. However, this approach presents its own set of challenges, notably the time-consuming nature of microscopic evaluation, taking over 15 min per slide. Additionally, the complexity of distinguishing between various *Plasmodium* species, exacerbated by their diverse life-cycle stages, poses a significant challenge. In response to these challenges, innovative solutions leveraging AI have emerged. CNNs, including models like YOLO, ResNet, and VGG, have been employed to streamline malaria diagnosis. These AI systems have demonstrated promising results, achieving accuracy, sensitivity, specificity, and F1 scores in the impressive range of 80–90% when compared to traditional LM. They prove effective not only in parasite classification but also in parasitemia estimation [100,101,102,103]. Several AI models have been developed for the automated detection of Plasmodium parasites, the causative agent of malaria, aiming to benefit affected regions. CNN with transfer learning is suggested for detecting and quantifying *Plasmodium falciparum* at different infection stages, where diagnostic accuracy heavily relies on microscopist expertise [104,105,106]. Multilayer perceptron and decision tree application to detect malaria parasites in full blood smear images achieved precision rates of 91.71% and 93.14% with large parasite sizes and 76.58% and 71.58% with small sizes [105]. Use of vision transformers (ViTs) to classify Plasmodium vivax life cycle states improved accuracy up to 90.03% [107]. Park et al. explored the application of AI in quantitative phase spectroscopy for the automated analysis of infected red blood cells, finding linear discriminant classification (LDC) most accurate (99.7%) for detecting schizont stage cells and k-nearest neighbor classification (NNC) slightly better (99.5%) than LDC (99.0%) or logistic regression (LR) (99.1%) for distinguishing late trophozoites from uninfected cells [108]. Hemachandran et al. utilized neural network models like CNN, MobileNetV2, and ResNet50 for automatic malaria blood smear diagnosis, with MobileNetV2 achieving 97.06% accuracy [106]. Additionally, point-of-care mobile digital microscopy and DL for detecting soil-transmitted helminths and Schistosoma haematobium have reported sensitivities ranging from 83.3% to 100% using sequential algorithms [109]. Incorporation of inexpensive mobile devices, such as smartphones and tablets, into the diagnostic process equipped with cameras attached to microscopes facilitate image acquisition and analysis. The integration of mobile technology, aided by the development of specific smartphone applications, has automated and accelerated the diagnostic process [110,111,112,113,114,115]. High-resolution cameras and the precision of CNNs has resulted in outstanding classification rates, with some instances achieving results within a mere 10 s [113,116] (Figure 3).

### 2.3. Mycobacteria

Mycobacterial infections pose a significant threat to global health, with approximately 10 million reported cases in 2018, leading to substantial morbidity and mortality. These infections primarily afflict individuals with underlying vulnerable conditions, such as immunodeficiency and malnutrition [117]. Within the mycobacterial group, organisms are generally classified into two categories: *Mycobacterium tuberculosis* and atypical mycobacteria. These microbes, characterized by small, bacilli-shaped organisms, present a diagnostic challenge, particularly with traditional LM and routine stains like H and E (hematoxylin and eosin). Despite efforts to enhance detection through special stains like Ziehl–Nielsen and Auramine O, the manual identification of mycobacteria in biological specimens remains a laborious process. Screening each slide can take 15–20 min, and the method is highly error prone. A positive diagnosis of mycobacteriosis typically relies on recognizing at least one reliably stained bacillus, with the predictive value associated with the number of acid-fast bacilli (AFB) present, which may be limited in early or incompletely resolved infections. In response to these challenges, there have been endeavors to automate AFB microscopic diagnosis using digital imaging and computer vision techniques. While cytology sputum smears were the primary focus in studies involving AI, a significant portion also explored CNNs on histological specimens like lung biopsies [118,119]. Image algorithms for AFB recognition have significantly improved, especially in sputum samples [120,121]. Technological advancements, including improved red versus green contrast in color-based pixel segmentation [122] and sophisticated AI algorithms [123], have significantly contributed to progress. A CNN algorithm based on whole-slide image (WSI) patch analysis showed superior accuracy [124], indicating that AI workflow could rule out negative cases without human review, streamlining diagnostics and reducing routine workloads.

Several early studies have proposed the use of CNNs for detecting and classifying *M. tuberculosis* [119,123,125,126]. Kuok et al. employed a refined faster region-based CNN (Faster R-CCN) model to automatically detect AFB in smear sputum slides, achieving an 86% detection rate compared to a SVM with a 70.93% detection rate [123]. In 2020, a study utilized a CNN-based active learning framework to identify mycobacteria in digitized Ziehl–Neelsen-stained human tissues, employing two CNN models, CNNIN and CNNAL. The study reported F1 scores of 99.03% and 98.75%, as well as 99.04% and 98.48% accuracy, respectively, for classifying microscopy slide images as AFB-positive and AFB-negative [125]. Advancements in digital imaging and analysis particularly through CNNs, offer promising avenues for automating the detection and classification of mycobacteria. Studies employing CNN models have demonstrated significant improvements in accuracy and efficiency, indicating a potential shift towards streamlined diagnostics and reduced workloads in the future.

## 3. Impact of AI in Revolutionizing Diagnostic Microbiology

### 3.1. Whole-Slide Imaging and Microbial Cytopathology

Cytology samples, in contrast to histology, exhibit a higher density of microorganisms and are more readily accessible in infected patients. It is important to highlight that, currently, WSI scanners face specific challenges when capturing images of cytopathology specimens compared to histological samples. This is primarily attributed to the need for digitizing cytology material on glass slides in multiple focal planes, employing techniques such as Z scanning to achieve optimal focus in the Z plane. However, this digitization process can significantly prolong both image acquisition and evaluation times [127,128,129]. Moreover, the distinctive nature of cytopathology introduces additional complexities. The results often generate large digital file sizes, necessitating extensive storage capacities and networks with substantial bandwidth [130]. This poses a considerable barrier to the widespread application of WSI for analyzing cytological preparations, especially in resource-limited regions where cost-effective solutions are crucial. In such contexts, the viability of WSI is further limited by the need for expensive infrastructure. In light of these challenges, alternative approaches, such as AI-based systems, emerge as promising solutions. These systems focus on analyzing static images acquired through relatively inexpensive portable devices, proving to be more suitable for routine use in resource-constrained settings. By circumventing the limitations associated with WSI, AI-based tools present a cost-effective and accessible option for the analysis of cytological samples, offering a practical alternative for regions where sophisticated imaging infrastructure is not readily available.

### 3.2. Detection and Characterization of Infectious Diseases

**Figure 4:**
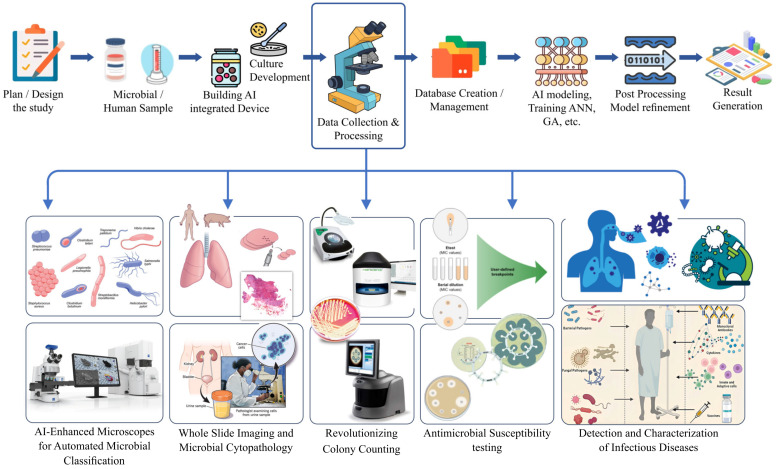
Artificial Intelligence (AI) is essential in identifying, categorizing, and studying the causes and effects of microbial diseases. AI systems utilize machine learning (ML) algorithms to examine extensive databases of microbiological samples, enabling the precise identification and classification of diseases based on their distinct properties. AI facilitates the automation of microbial colony counts, resulting in time savings and less errors in laboratory procedures. Furthermore, systems driven by AI have the ability to ascertain antibiotic susceptibility, thereby providing guidance for the implementation of appropriate treatment options. In addition, AI aids in the identification of microbial infections by evaluating genomic data and recognizing patterns that indicate virulence or antibiotic resistance. This allows for more precise therapies to be implemented.

AI has revolutionized pathology by automating the recognition of morphological patterns and cytologic changes associated with infections, rather than focusing solely on microorganism detection. Image analysis, exemplified in a recent study [131], shows significant concordance with WSI and LM in distinguishing *Helicobacter pylori*-related gastritis and autoimmune gastritis. Integration of DL algorithms has led to sophisticated tools for discerning nuclear features, achieving a remarkable 92.7% accuracy in predicting HPV status in cervical biopsies with an average analysis time of 22 s [132]. This highlights the potential of AI in assisting pathologists by identifying crucial nuclear texture and shape features. The synergy of digital pathology and AI has transformed the evaluation of pathology specimens, with studies consistently showing results comparable to the gold standard of human examination using LM. As AI-based tools evolve and become more accessible, they emerge as potent instruments in addressing the threat posed by infectious diseases, both known and emerging. Of particular note is the potential impact of AI-based systems in resource-limited countries, where the burden of infectious diseases is high, and there is a shortage of skilled laboratorians and equipment. The adoption of these systems in such settings could prove exceptionally beneficial, offering a scalable and efficient solution to enhance diagnostic capabilities and contribute to effective disease management. The intersection of AI and pathology holds promise for improving global healthcare, especially in regions where traditional resources are constrained (Figure 4).

### 3.3. AI-Enhanced Microscopes for Automated Microbial Classification

Microscopes integrated with AI exhibit significant potential in assisting microbiologists in the examination of organisms and leveraging data for diagnostic purposes and root cause analysis. An earlier study showed the efficacy of an automated AI-enhanced microscope system in swiftly and accurately identifying bacterial images [133]. The research employed an automated microscope specifically designed for capturing high-resolution image data from microscopic slides. Utilizing a CNN, an AI model inspired by the mammalian visual cortex, the study focused on assessing and categorizing bacteria based on their morphology. This encompassed identifying common bacterial shapes such as rod- or coccus-shaped bacteria, a hallmark of *Staphylococcus* species, and pairs or chains, indicating *Streptococcus* species. The training process adopted a gradual approach, commencing with an unschooled neural network that analyzed over 25,000 images from various samples. Through the cropping of these images, researchers generated more than 100,000 training images, each containing previously identified bacteria. The machine intelligence progressively acquired the capability to classify images into three distinct categories (rod-shaped, round clusters, and round chains or pairs), achieving an impressive accuracy rate of almost 95 percent upon the completion of the training phase. In the subsequent phase, the algorithm was put to the test by autonomously sorting new images from 189 slides without human intervention, achieving an overall accuracy rate of 93 percent across all three categories. The findings of the study suggest that with further refinement and training, this AI-enhanced platform could potentially evolve into a fully automated classification system, providing valuable diagnostic capabilities. Additionally, the system’s functionality enables the remote transmission of images to microbiologists globally, fostering enhanced collaboration and accessibility in the field of microbiology [133] (Figure 4).

### 3.4. Revolutionizing Colony Counting

The process of colony counting, seemingly simple at first glance, is not without its challenges. Human error, whether arising from visual oversight or fatigue, can introduce inaccuracies into the counting process [134]. However, recent strides in visual assessments, particularly leveraging ML, have given rise to high-resolution image analysis systems that exhibit heightened sensitivity. These advanced systems excel in identifying small and mixed colonies that may escape the human eye. For the development of an efficient, automated, and AI-driven colony counting system, certain key functionalities are imperative [135]. This includes the need for standardized and accurate results, taking into account various colony parameters such as size, shape, contrast, and instances of overlapping. Automatic colony separation, especially in cases of closely situated colonies, is essential. Accurate colony counting, optimal image acquisition performance, effective resolution, data management, and visualization of both white light and fluorescent colonies are crucial [136]. The system must differentiate chromatic and achromatic images, handle aggregated colonies, count the entire plate or specific sectors, and provide results within one second per plate. Additional features include a real-time, full-color, on-screen display; a zoom function for detailed examination; and software for seamless data collection, analysis, and transfer to a laboratory information management system (LIMS) [137]. In lab settings, AI efficiently processes bulk samples like urine, crucial for detecting UTIs and bacterial infections, especially in females. In fully automated labs, software algorithms, notably APAS Independence, cleared by the FDA, differentiate negative urine cultures from growth beyond the defined threshold [138]. APAS aids in excluding cultures with no growth and is valuable for MRSA detection. PhenoMatrix from bioMérieux has been studied for identifying group B *Streptococcus* and *Streptococcus pyogenes* [139]. 

Additionally, Becton Dickinson Kiestra has developed a deep CNN that captures images of urine samples for culture analysis [140]. These advancements signify significant progress in automating and enhancing the efficiency of laboratory processes, particularly in the analysis of samples susceptible to bulk processing [138,139]. Recent studies underscore the potential of expert image analysis systems in analyzing culture plates, offering a screening technique for rapid and efficient differentiation of positive and negative cultures [141,142]. This approach facilitates the exclusion of cultures showing no growth or contamination with normal flora from further analysis and processing, leading to a reduction in manual workload and enhanced utilization of human resources. However, it is essential to acknowledge the challenges associated with the accurate assessment of contamination by AI systems. This task is inherently complex and may not be entirely achievable due to various influencing factors, including the type of specimen, transit and processing time, patient history, clinical presentation, and the expert judgment of microbiologists [139]. Consequently, expecting an AI system to perform such an extensive analysis presents inherent difficulties. This critical evaluation explores the potential and limitations of integrating expert image analysis systems into microbiological culture plate analysis. While ML applications for colony counting continue to advance, challenges persist [143]. Issues such as low image resolution, high colony-forming unit (CFU) density, background noise, artifacts on the dish boundary, and proximity of CFUs to the dish’s edge are hurdles being addressed through iterative learning patterns. ML approaches also extend to other types of image analysis, including applications in Gram stains (Figure 4).

### 3.5. Advancements in AI Applications for Antimicrobial Susceptibility Testing

Recent advancements in research underscore the pivotal role that AI plays in the field of diagnosing pathogenic microorganisms and evaluating their antimicrobial susceptibility patterns [144]. AI algorithms have proven their efficacy in the detection of resistance to aminoglycosides within bacteria such as *Escherichia coli* and *Staphylococcus aureus* [144]. Additionally, the application of computer vision technology to screening cultures has facilitated the early identification of drug-resistant bugs, including vancomycin-resistant Enterococcus (VRE) or MRSA, thereby allowing for timely intervention in terms of treatment and control [145].

The integration of AI principles into total laboratory automation is gaining prominence, particularly in large-scale laboratories that specialize in microbial identification and antimicrobial susceptibility testing. Remarkable systems, such as the Kiestra TLA system by Becton Dickinson in Franklin Lakes, NJ, USA, and WASPLab by Copan Diagnostics Inc. in Murrieta, CA, USA, exemplify the increasing prevalence of AI in these settings [146]. MALDI-TOF MS has played a crucial role in microbial diagnostics over the past decade. The fusion of MALDI-TOF MS with AI and ML has substantially enhanced its diagnostic capabilities, extending beyond microorganism identification to predicting AMR. Recent research demonstrated the effectiveness of combining MALDI-TOF MS with ML analysis, utilizing the Python programming language. This approach proved successful in detecting AMR in *Campylobacter* species with a maximum sensitivity and precision of 92.3% and 81.2%, respectively [147]. These advancements highlight the immense potential of AI to revolutionize microbial diagnostics and contribute to the development of more effective treatment strategies (Figure 4).

## 4. Conclusions: Challenges and Future Directions

AI-powered diagnostic tools, driven by ML and computational progress, are leading a healthcare revolution [148]. They swiftly identify infections, including novel and drug-resistant strains, contributing significantly to public health responses [149]. Ongoing technological advancements enable individuals to actively monitor their health, facilitating early identification of microbial diseases [148]. AI’s impact in healthcare extends to vaccine development, AMR, and epidemiological surveillance, marking a paradigm shift [150]. Its incorporation in microbial diagnosis is a cornerstone for the healthcare sector’s transformative journey. As ethical and regulatory frameworks progress, AI in microbial diagnosis is poised to deliver personalized and accurate solutions, ensuring patient privacy and equitable access [151]. AI solidifies its role as a key player in shaping the future of healthcare, revolutionizing delivery while upholding ethical standards and accessibility [152].

In reviewing studies on AI in diagnostic microbiology, it is observed that many face limitations, including the absence of external datasets for validating algorithms in clinical settings. The scarcity of publicly available datasets for developing DL systems poses challenges in result validation. The reliance on supervised or semi-supervised methods in current DL approaches, which require expert image annotation, is a further limitation. Addressing these issues calls for technological innovation to increase the number of CNNs and access to more widely available databases. Despite these challenges, the potential benefits of AI in diagnostic microbiology are evident.

AI also faces challenges in interpreting single-organism polymorphisms and accurately recognizing contamination, requiring years of experience and comprehensive clinical context understanding. Further, the interpretation of antimicrobial susceptibility testing data is hindered by diverse mechanisms of action and coexisting resistances. Creating fixed programs and standardized algorithms for predicting organism susceptibility to antimicrobials is challenging due to overlapping mechanisms and the synergistic actions of antibiotics, along with imperfect data.

The integration of AI in diagnostic microbiology laboratories is seen as inevitable. To facilitate this transition, there is a need for the development of more robust AI products and algorithms to ensure stability and user-friendliness. This would not only reduce expenses and turnaround time but also minimize errors, benefiting both patients and medical staff. Efforts should be directed towards enhancing education and training on AI-based technologies for technicians, extending these technologies to peripheral and remote areas. Overall, the integration of AI in diagnostic microbiology holds the promise of transformative advancements with proper navigation of the current challenges.

While AI has the potential to revolutionize diagnostic microbiology by improving microbial identification accuracy and predicting antimicrobial susceptibility, its integration into microbiology laboratories face many challenges due to the technology being in its early development phase. Addressing limitations such as potential misinterpretation of organism polymorphisms, difficulty in distinguishing pathogens from contaminants, and the absence of fixed algorithms for integrating multiple drug and resistance mechanisms is crucial before AI becomes a routine part of microbiology laboratories. Although AI offers quick and accurate diagnoses and treatment predictions, it should only partially replace human intervention in the microbiology laboratory.

## Figures and Tables

**Table 1:** Overview of AI Applications in COVID-19 Diagnosis and Detection.

Study	AI Model/Method	Performance (Sensitivity and Specificity)
Alouani et al. [71]	Deep learning model (qPCRdeepNet)	Improved specificity of RT-PCR test for SARS-CoV-2 through deep convolutional neural network analysis of fluorescent readings
Lee et al. [72]	Deep learning model (LSTM)	Shortened RT-PCR diagnosis time using raw fluorescence data in each cycle, integrated with patient clinical characteristics, blood test results, and chest CT imaging data
AI-based detection system [73]	AI-based detection and classification system	Automated categorization of RT-PCR data (positive, weak-positive, negative, or re-run)
Villarreal-González et al. [74]	Various ML models	Detection of atypical RT-PCR profiles, reducing false positives
Alvargonzález et al. [75]	ML algorithm based on Ct values	Distinguishable patterns in Ct values aiding detection of SARS-CoV-2 variants
Beduk et al. [76]	Dense Neural Network algorithm	Detection of SARS-CoV-2 variants using laser-scribed graphene sensors coupled with gold nanoparticles biosensing platform
Tschoellitsch et al. [77]	Random Forest algorithm	Prediction of RT-PCR test results using routine blood test data
Brinati et al. [78]	ML classification models using hemato-chemical values	Detection of COVID-19 infection with high accuracy and sensitivity using routine blood exam data
Yang et al. [79]	ML model	Identification of useful routine blood parameters for COVID-19 diagnosis
Abayomi-Alli et al. [93]	Ensemble learning approach with 15 supervised ML algorithms	Effective detection of COVID-19 using routine laboratory blood test results
MALDI-TOF-MS combined with ML [22]	MALDI-TOF-MS combined with ML algorithms	Detection of COVID-19 protein profiles in nasopharyngeal swab samples
Rocca et al. [80]	LC/MS-MS combined with ML	Discrimination of SARS-CoV-2-positive and negative patients through targeted plasma metabolomics
Rosado et al. [82]	Various ML approaches	Identification of serological signatures of SARS-CoV-2 infection
Detection in saliva, nasal swabs, plasma, and serum samples [94,95,96,97]	Various techniques (MALDI-TOF-MS, SERS, LC-MS, MALDI-MS)	Detection of SARS-CoV-2 in various biological samples
Apostolopoulos et al. [84]	Deep learning with transfer learning	Extraction of COVID-19 biomarkers from CX-R images
Singh et al. [85]	Convolutional Neural Networks with multi-objective MODE	Efficient classification of COVID-19-infected patients using chest CT images
Mei et al. [86]	AI algorithms integrating chest CT findings with clinical data	Rapid diagnosis of COVID-19 positive patients by integrating chest CT findings with clinical symptoms, exposure history, and laboratory testing
Systematic review [87,88]	Various deep learning models	High performance of deep learning models in interpreting chest CT scans for COVID-19 diagnosis
Wang et al. [91]	Deep learning models (Resnet, Densenet, VGG, Mobilenet, etc.)	Pooled sensitivity, specificity, AUROC, and diagnostic odds ratio for COVID-19 diagnosis using chest CT scans
Ozsahin et al. [92]	AI techniques for CT imaging	High sensitivity, specificity, precision, accuracy, AUROC, and F1 score for COVID-19 diagnosis using CT scans

## Data Availability

No new data were created or analyzed in this study.
